# Bis{tris­[3-(2-pyrid­yl)-1*H*-pyrazole]iron(II)} tetra­deca­molybdo(V,VI)silicate

**DOI:** 10.1107/S1600536810002412

**Published:** 2010-01-23

**Authors:** Peihai Wei, Dong Yuan, Wencai Zhu, Xiutang Zhang, Bo Hu

**Affiliations:** a Advanced Material Institute of Research, Department of Chemistry and Chemical Engineering, ShanDong Institute of Education, Jinan 250013, People’s Republic of China; b College of Chemistry and Chemical Engineering, Liaocheng University, Liaocheng 252059, People’s Republic of China

## Abstract

The asymmetric unit of the title compound, [Fe(C_8_H_7_N_3_)_3_]_2_[SiMo_14_O_44_], consists of a complex [Fe(C_8_H_7_N_3_)_3_]^2+^ cation and half of a derivative of an α-Keggin-type anion, [SiMo_14_O_44_]^4−^. In the mixed-valent Mo^V/VI^ anion, the α-Keggin type core is capped on two oppositely disposed tetra­gonal faces by additional (MoO_2_) units. The [SiMo_14_O_44_]^4−^ anion shows disorder. Two O atoms of the central SiO_4_ group (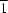 symmetry) are equally disordered about an inversion centre. Moreover, two of the outer bridging O atoms and the O atoms of the capping (MoO_2_) unit are likewise disordered. The Fe^2+^ cation is surrounded in a slightly distorted octa­hedral coordination by six N atoms from three 3-(2-pyrid­yl)-1*H*-pyrazole ligands. N—H⋯O hydrogen bonding between the cations and anions leads to a consolidation of the structure.

## Related literature

For general background to polyoxometalates, see: Pope & Müller (1991 ▶). For polyoxometalates modified with amines, see: Zhang, Dou *et al.* (2009 ▶); Zhang, Wei, Shi *et al.* (2010*a* ▶,*b* ▶); Zhang, Wei, Sun *et al.* (2009 ▶); Zhang, Wei, Zhu *et al.* (2010 ▶); Zhang, Yuan *et al.* (2010 ▶). For another structure containing the α-Keggin-type derivative [SiMo_14_O_44_]^4−^, see: Dolbecq *et al.* (1999 ▶). For background to the bond-valence method, see: Brese & O’Keeffe (1991 ▶). For the role of amines in hydro­thermal synthesis, see: Yang *et al.* (2003 ▶).
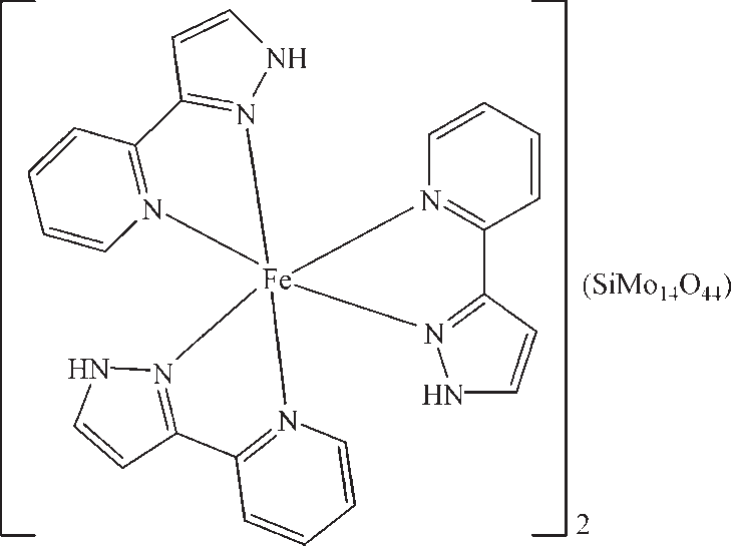


## Experimental

### 

#### Crystal data

[Fe(C_8_H_7_N_3_)_3_]_2_[SiMo_14_O_44_]
*M* *_r_* = 3057.95
Monoclinic, 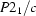

*a* = 13.055 (3) Å
*b* = 16.931 (3) Å
*c* = 18.562 (4) Å
β = 102.69 (3)°
*V* = 4002.6 (14) Å^3^
*Z* = 2
Mo *K*α radiation
μ = 2.58 mm^−1^
*T* = 293 K
0.12 × 0.10 × 0.08 mm

#### Data collection

Bruker APEXII CCD diffractometer
Absorption correction: multi-scan (*SADABS*; Bruker, 2001 ▶) *T*_min_ = 0.747, *T*_max_ = 0.820
27223 measured reflections
7026 independent reflections
4730 reflections with *I* > 2σ(*I*)
*R*_int_ = 0.076

#### Refinement

*R*[*F* ^2^ > 2σ(*F* ^2^)] = 0.059
*wR*(*F* ^2^) = 0.157
*S* = 1.00
7026 reflections
638 parameters
444 restraints
H-atom parameters constrained
Δρ_max_ = 1.47 e Å^−3^
Δρ_min_ = −1.52 e Å^−3^

### 

Data collection: *APEX2* (Bruker, 2004 ▶); cell refinement: *SAINT-Plus* (Bruker, 2001 ▶); data reduction: *SAINT-Plus*; program(s) used to solve structure: *SHELXS97* (Sheldrick, 2008 ▶); program(s) used to refine structure: *SHELXL97* (Sheldrick, 2008 ▶); molecular graphics: *SHELXTL* (Sheldrick, 2008 ▶); software used to prepare material for publication: *SHELXTL*.

## Supplementary Material

Crystal structure: contains datablocks global, I. DOI: 10.1107/S1600536810002412/wm2300sup1.cif

Structure factors: contains datablocks I. DOI: 10.1107/S1600536810002412/wm2300Isup2.hkl

Additional supplementary materials:  crystallographic information; 3D view; checkCIF report

## Figures and Tables

**Table 1:** Selected bond lengths (Å)

Si1—O17*A*	1.606 (13)
Si1—O18*A*	1.648 (13)
Si1—O18*B*	1.670 (13)
Si1—O17*B*	1.684 (13)
Fe1—N2	2.105 (11)
Fe1—N8	2.119 (11)
Fe1—N5	2.153 (11)
Fe1—N3	2.173 (11)
Fe1—N9	2.186 (12)
Fe1—N6	2.206 (11)

**Table 2:** Hydrogen-bond geometry (Å, °)

*D*—H⋯*A*	*D*—H	H⋯*A*	*D*⋯*A*	*D*—H⋯*A*
N4—H4⋯O13^i^	0.86	2.15	2.973 (17)	159
N1—H1*A*⋯O21*A*^ii^	0.86	2.17	2.96 (3)	152
N1—H1*A*⋯O21^ii^	0.86	2.05	2.844 (19)	153

Symmetry codes: (i) 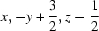; (ii) 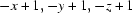.

**Fig. 1.**
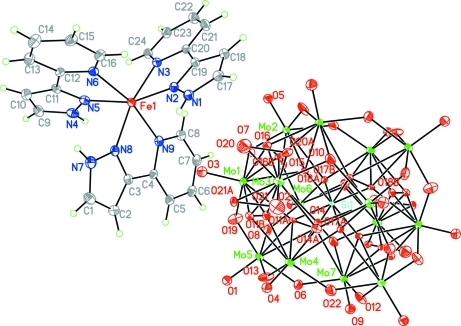
The building blocks of the title compound with the atom-labelling scheme. Displacement ellipsoids are drawn at the 30% probability level; H atoms are given as spheres of arbitrary radius.

**Fig. 2.**
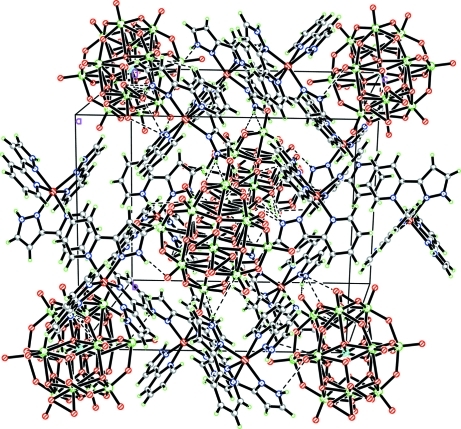
The crystal packing of the title compound, displayed with hydrogen bonds as dashed lines.

## supplementary crystallographic information

### Comment

The design and synthesis of polyoxometalates has attracted continuous research interest not only because of their appealing structural and topological novelties, but also due to their interesting optical, electronic, magnetic, and catalytic properties, as well as their potential medical applications (Pope & Müller, 1991). In our research group, organic amines, such as 3-(2-pyridyl)pyrazole and pyrazine, are used to effectively modify polyoxomolybdates under hydrothermal condictions (Zhang, Dou *et al.*, 2009; Zhang, Wei, Shi *et al.*, 2010*a*,*b*; Zhang, Wei, Sun *et al.*, 2009; Zhang, Wei, Zhu *et al.*, 2010; Zhang, Yuan *et al.*, 2010). Here, we describe the synthesis and structural characterization of the title compound.

As shown in Figure 1, the title compound consists of two subunits, *viz.* of a complex caion [Fe(C_8_H_7_N_3_)_3_]^2+^ and a derivative of the α-Keggin-type anion with overall composition [SiMo_14_O_44_]^4-^. The iron(II) ion is in a distorted octahedral coordination by six N atoms from two 3-(2-pyridyl)pyrazole ligands. The Fe—N bond lengths are in the range of 2.105 (11)—2.206 (11) Å. The anion [SiMo_14_O_44_]^4-^ can be described as a derivative of an α-Keggin-type core capped on two oppositely disposed tetragonal faces by (MoO_2_) subunits (Dolbecq *et al.*, 1999). The molybdenum atoms of the disordered capping unit have a highly distorted coordination environment with short Mo—Ot bonds [1.70 (15)–1.84 (3) Å] and longer Mo—O bridging bonds [1.998 (16)–2.174 (15) Å].

The formula sum of the title compound reveals that the valence of some metal atoms in the title compound are (partly) reduced (part of Mo^6+^ to Mo^5+^; Fe^3+^ to Fe^2+^). The employed organic ligand appears to adjust the pH value, and additionally supplies reducing electrons, which is a commonly observed feature of hydrothermal syntheses when organic amines are used to prepare various hybrid materials, zeolites or metal phosphates (Yang *et al.*, 2003). The oxidation states of the metals are confirmed by bond valence sum calculations (Brese & O'Keeffe, 1991). For the Mo and Fe atoms in the title compound the bond valence sums equal to 5.93, 4.95, 5.83, 6.17, 5.81, 6.18, 5.24, and 2.01.

N—H···O hydrogen bonding between the cations and anions leads to a consolidation of the structure (Fig. 2; Table 2).

### Experimental

A mixture of 3-(2-pyridyl)pyrazole (1 mmoL 0.14 g), sodium molybdate (2 mmoL, 0.48 g), sodium silicate nonahydrate (0.2 mmoL, 0.05 g) and iron(III) chloride hexahydrate (0.25 mmol, 0.06 g) in 10 ml distilled water was sealed in a 25 ml Teflon-lined stainless steel autoclave and was kept at 433 K for three days. Brown crystals suitable for the X-ray experiment were obtained. Anal. C_48_H_42_Fe_2_Mo_14_N_18_O_44_Si: C, 18.85; H, 1.38; N, 8.25. Found: C, 18.75; H, 1.12; N, 8.15%.

The TGA measure,ent shows that the release of organic liangds takes place above *ca* 623 K. The overall thermal decomposition process can be described by the following equation: C_48_H_42_Fe_2_Mo_14_N_18_O_44_Si + 84O_2_ = 21H_2_O + 48CO_2_ + 9N_2_O_5_ + Fe_2_O_3_ + SiO_2_ + 14MoO_3_ Eq.(1). IR bands appear at the following wavelengths (cm^-1^): 3303, 3117, 1697, 1604, 1550, 1485, 1355, 1300, 1171, 1088, 1051, 903, 765, 664, 507, 451, 414.

### Refinement

All hydrogen atoms bound to aromatic carbon atoms were refined in calculated positions using a riding model with a C—H distance of 0.93 Å and *U*_iso_ = 1.2*U*_eq_(C). Hydrogen atoms attached to aromatic N atoms were refined with a N—H distance of 0.86 Å and *U*_iso_ = 1.2*U*_eq_(N). In the SiO_4_ unit, the two oxygen atoms (O17 and O18) are equally disordered about the inversion centre. Four O atoms (O11, O16, O20, O21) are also disordered and were refined with split positions and an occupancy ratio of 1:1 (O 11, 16) and 3:1 (O20, 21). In the final difference Fourier map the highest peak is 1.25 Å from atom O20A and the deepest hole is 0.67 Å from atom Mo3. The highest peak is located in the voids of the crystal structure and may be associated with an additional water molecule. However, refinement of this position did not result in a reasonable model. Hence this position was also excluded from the final refinement.

### Crystal data

**Table:**

[Fe(C_8_H_7_N_3_)_3_]_2_[SiMo_14_O_44_]	*F*(000) = 2924
*M**_r_* = 3057.95	*D*_x_ = 2.537 Mg m^−^^3^
Monoclinic, *P*2_1_/*c*	Mo *K*α radiation, λ = 0.71073 Å
Hall symbol: -P 2ybc	Cell parameters from 7026 reflections
*a* = 13.055 (3) Å	θ = 1.7–25.0°
*b* = 16.931 (3) Å	µ = 2.58 mm^−^^1^
*c* = 18.562 (4) Å	*T* = 293 K
β = 102.69 (3)°	Block, brown
*V* = 4002.6 (14) Å^3^	0.12 × 0.10 × 0.08 mm
*Z* = 2	

### Data collection

**Table:**

Bruker APEXII CCD diffractometer	7026 independent reflections
Radiation source: fine-focus sealed tube	4730 reflections with *I* > 2σ(*I*)
graphite	*R*_int_ = 0.076
phi and ω scans	θ_max_ = 25.0°, θ_min_ = 1.7°
Absorption correction: multi-scan (*SADABS*; Bruker, 2001)	*h* = −15→15
*T*_min_ = 0.747, *T*_max_ = 0.820	*k* = −20→20
27223 measured reflections	*l* = −21→22

### Refinement

**Table:**

Refinement on *F*^2^	Primary atom site location: structure-invariant direct methods
Least-squares matrix: full	Secondary atom site location: difference Fourier map
*R*[*F*^2^ > 2σ(*F*^2^)] = 0.059	Hydrogen site location: inferred from neighbouring sites
*wR*(*F*^2^) = 0.157	H-atom parameters constrained
*S* = 1.00	*w* = 1/[σ^2^(*F*_o_^2^) + (0.058*P*)^2^ + 47.4341*P*] where *P* = (*F*_o_^2^ + 2*F*_c_^2^)/3
7026 reflections	(Δ/σ)_max_ = 0.001
638 parameters	Δρ_max_ = 1.47 e Å^−^^3^
444 restraints	Δρ_min_ = −1.51 e Å^−^^3^

### Special details

**Table:**

Geometry. All e.s.d.'s (except the e.s.d. in the dihedral angle between two l.s. planes) are estimated using the full covariance matrix. The cell e.s.d.'s are taken into account individually in the estimation of e.s.d.'s in distances, angles and torsion angles; correlations between e.s.d.'s in cell parameters are only used when they are defined by crystal symmetry. An approximate (isotropic) treatment of cell e.s.d.'s is used for estimating e.s.d.'s involving l.s. planes.
Refinement. Refinement of *F*^2^ against ALL reflections. The weighted *R*-factor *wR* and goodness of fit *S* are based on *F*^2^, conventional *R*-factors *R* are based on *F*, with *F* set to zero for negative *F*^2^. The threshold expression of *F*^2^ > σ(*F*^2^) is used only for calculating *R*-factors(gt) *etc*. and is not relevant to the choice of reflections for refinement. *R*-factors based on *F*^2^ are statistically about twice as large as those based on *F*, and *R*- factors based on ALL data will be even larger.

### Fractional atomic coordinates and isotropic or equivalent isotropic displacement parameters (Å^2^)

**Table:**

	*x*	*y*	*z*	*U*_iso_*/*U*_eq_	Occ. (<1)
Si1	0.0000	0.5000	0.5000	0.0193 (8)	
Fe1	0.36074 (14)	0.58896 (11)	0.17093 (11)	0.0497 (5)	
Mo1	0.20889 (7)	0.55765 (6)	0.41647 (5)	0.0364 (3)	
Mo2	−0.00697 (9)	0.46521 (7)	0.30342 (6)	0.0431 (3)	
Mo3	0.33482 (8)	0.44820 (7)	0.54989 (6)	0.0464 (3)	
Mo4	0.21554 (8)	0.59444 (6)	0.61448 (6)	0.0408 (3)	
Mo5	0.05628 (8)	0.70722 (6)	0.47289 (6)	0.0389 (3)	
Mo6	−0.16040 (7)	0.61555 (6)	0.35957 (6)	0.0378 (3)	
Mo7	−0.14918 (7)	0.65270 (6)	0.55885 (5)	0.0339 (3)	
C1	0.5226 (13)	0.7541 (10)	0.3320 (9)	0.080 (5)	
H1	0.5807	0.7807	0.3592	0.096*	
C2	0.4230 (13)	0.7709 (10)	0.3329 (9)	0.074 (4)	
H2	0.3975	0.8084	0.3611	0.088*	
C3	0.3659 (11)	0.7178 (8)	0.2806 (7)	0.055 (3)	
C4	0.2536 (10)	0.7064 (8)	0.2539 (7)	0.048 (3)	
C5	0.1772 (11)	0.7481 (9)	0.2789 (8)	0.060 (4)	
H5	0.1956	0.7867	0.3151	0.072*	
C6	0.0709 (12)	0.7310 (10)	0.2481 (9)	0.069 (4)	
H6	0.0182	0.7576	0.2648	0.082*	
C7	0.0462 (12)	0.6771 (10)	0.1956 (9)	0.070 (4)	
H7	−0.0240	0.6661	0.1752	0.084*	
C8	0.1236 (11)	0.6369 (9)	0.1708 (8)	0.063 (4)	
H8	0.1044	0.5995	0.1336	0.076*	
C9	0.4257 (14)	0.7723 (10)	0.0181 (9)	0.079 (5)	
H9	0.4153	0.8194	−0.0084	0.095*	
C10	0.5155 (13)	0.7282 (10)	0.0337 (8)	0.072 (4)	
H10	0.5774	0.7377	0.0181	0.086*	
C11	0.4943 (11)	0.6643 (8)	0.0791 (7)	0.053 (3)	
C12	0.5598 (10)	0.6004 (8)	0.1139 (7)	0.048 (3)	
C13	0.6664 (12)	0.5936 (10)	0.1094 (8)	0.072 (4)	
H13	0.6971	0.6311	0.0843	0.087*	
C14	0.7234 (15)	0.5302 (12)	0.1431 (10)	0.095 (6)	
H14	0.7922	0.5218	0.1388	0.114*	
C15	0.6750 (13)	0.4797 (10)	0.1834 (9)	0.076 (5)	
H15	0.7126	0.4373	0.2077	0.091*	
C16	0.5731 (12)	0.4904 (9)	0.1886 (8)	0.067 (4)	
H16	0.5430	0.4553	0.2164	0.081*	
C17	0.3580 (12)	0.3850 (9)	0.3102 (9)	0.065 (4)	
H17	0.3773	0.3553	0.3532	0.078*	
C18	0.2902 (12)	0.3614 (9)	0.2482 (8)	0.066 (4)	
H18	0.2535	0.3141	0.2391	0.079*	
C19	0.2886 (10)	0.4280 (8)	0.1993 (8)	0.054 (3)	
C20	0.2335 (10)	0.4416 (8)	0.1235 (7)	0.052 (3)	
C21	0.1621 (13)	0.3881 (10)	0.0809 (10)	0.081 (5)	
H21	0.1494	0.3387	0.0989	0.098*	
C22	0.1117 (13)	0.4130 (11)	0.0105 (9)	0.081 (5)	
H22	0.0637	0.3795	−0.0191	0.097*	
C23	0.1311 (13)	0.4857 (10)	−0.0161 (9)	0.075 (4)	
H23	0.0938	0.5025	−0.0621	0.090*	
C24	0.2060 (11)	0.5332 (9)	0.0258 (8)	0.059 (4)	
H24	0.2231	0.5807	0.0062	0.070*	
N1	0.3925 (9)	0.4562 (7)	0.3005 (6)	0.060 (3)	
H1A	0.4368	0.4813	0.3338	0.072*	
N2	0.3507 (8)	0.4859 (6)	0.2324 (6)	0.051 (3)	
N3	0.2551 (9)	0.5129 (7)	0.0943 (6)	0.057 (3)	
N4	0.3541 (10)	0.7337 (7)	0.0492 (7)	0.072 (4)	
H4	0.2902	0.7490	0.0449	0.087*	
N5	0.3958 (9)	0.6673 (6)	0.0883 (6)	0.055 (3)	
N6	0.5155 (8)	0.5512 (6)	0.1541 (6)	0.052 (3)	
N7	0.5271 (10)	0.6937 (8)	0.2864 (7)	0.072 (4)	
H7A	0.5839	0.6725	0.2793	0.086*	
N8	0.4298 (8)	0.6712 (7)	0.2534 (6)	0.052 (3)	
N9	0.2279 (9)	0.6507 (7)	0.1994 (6)	0.058 (3)	
O1	0.0848 (7)	0.8012 (5)	0.4612 (5)	0.058 (2)	
O2	−0.2484 (7)	0.6655 (6)	0.2974 (5)	0.061 (2)	
O3	0.3156 (7)	0.5797 (6)	0.3861 (5)	0.061 (2)	
O4	0.3275 (6)	0.6295 (5)	0.6651 (5)	0.052 (2)	
O5	−0.0141 (7)	0.4491 (5)	0.2150 (5)	0.054 (2)	
O6	−0.0494 (8)	0.7163 (6)	0.5323 (5)	0.071 (3)	
O7	0.1182 (8)	0.5280 (6)	0.3314 (5)	0.074 (3)	
O8	−0.0581 (7)	0.6902 (6)	0.3890 (5)	0.071 (3)	
O9	−0.2306 (7)	0.7206 (6)	0.5792 (5)	0.066 (3)	
O10	0.2238 (9)	0.3640 (6)	0.5498 (5)	0.080 (3)	
O11A	0.2488 (15)	0.5526 (11)	0.5273 (10)	0.040 (5)	0.50
O11B	0.2997 (13)	0.5646 (10)	0.5258 (9)	0.033 (4)	0.50
O12	−0.0731 (8)	0.6245 (6)	0.6494 (5)	0.076 (3)	
O13	0.1592 (8)	0.6844 (6)	0.5658 (5)	0.077 (3)	
O14	0.2189 (12)	0.4826 (9)	0.6113 (9)	0.033 (4)	0.50
O14A	0.2767 (14)	0.4726 (10)	0.6443 (9)	0.043 (4)	0.50
O15	−0.0919 (9)	0.5605 (6)	0.2994 (7)	0.090 (3)	
O16	0.2659 (13)	0.4376 (10)	0.4430 (9)	0.036 (4)	0.50
O16B	0.2127 (12)	0.4516 (9)	0.4492 (8)	0.028 (4)	0.50
O17A	−0.0469 (10)	0.5874 (8)	0.4826 (7)	0.029 (3)	0.50
O17B	−0.0747 (10)	0.4992 (8)	0.4136 (7)	0.028 (3)	0.50
O18A	0.0684 (10)	0.4742 (8)	0.4390 (7)	0.027 (3)	0.50
O18B	−0.0905 (10)	0.4306 (8)	0.5014 (7)	0.028 (3)	0.50
O19	0.1508 (9)	0.6552 (7)	0.4225 (6)	0.087 (3)	
O20	0.4195 (13)	0.4254 (10)	0.4906 (10)	0.104 (5)	0.75
O20A	0.412 (2)	0.3654 (19)	0.5690 (16)	0.041 (8)	0.25
O21A	0.449 (2)	0.5140 (17)	0.5616 (15)	0.034 (7)	0.25
O21	0.4286 (12)	0.4547 (10)	0.6293 (9)	0.100 (5)	0.75
O22	0.1328 (8)	0.5930 (6)	0.6805 (7)	0.085 (3)	

### Atomic displacement parameters (Å^2^)

**Table:**

	*U*^11^	*U*^22^	*U*^33^	*U*^12^	*U*^13^	*U*^23^
Si1	0.0201 (18)	0.0165 (19)	0.0221 (18)	0.0002 (15)	0.0064 (15)	−0.0006 (15)
Fe1	0.0472 (11)	0.0411 (11)	0.0604 (12)	−0.0018 (9)	0.0109 (9)	−0.0033 (9)
Mo1	0.0292 (5)	0.0435 (6)	0.0374 (6)	−0.0003 (4)	0.0090 (4)	0.0018 (5)
Mo2	0.0549 (7)	0.0418 (7)	0.0353 (6)	−0.0056 (5)	0.0162 (5)	−0.0055 (5)
Mo3	0.0418 (6)	0.0427 (7)	0.0500 (7)	0.0061 (5)	−0.0001 (5)	−0.0015 (5)
Mo4	0.0297 (5)	0.0392 (6)	0.0513 (7)	−0.0034 (4)	0.0043 (4)	−0.0140 (5)
Mo5	0.0460 (6)	0.0235 (5)	0.0442 (6)	0.0013 (4)	0.0036 (5)	0.0015 (5)
Mo6	0.0300 (5)	0.0322 (6)	0.0487 (6)	0.0053 (4)	0.0030 (4)	0.0082 (5)
Mo7	0.0296 (5)	0.0275 (5)	0.0440 (6)	0.0044 (4)	0.0071 (4)	−0.0050 (4)
C1	0.075 (8)	0.079 (8)	0.079 (8)	−0.011 (7)	0.001 (7)	−0.005 (7)
C2	0.080 (8)	0.072 (8)	0.072 (8)	−0.004 (7)	0.024 (7)	−0.010 (7)
C3	0.053 (7)	0.053 (7)	0.058 (7)	0.000 (6)	0.007 (6)	0.011 (6)
C4	0.051 (6)	0.045 (6)	0.051 (6)	0.010 (5)	0.017 (5)	0.015 (5)
C5	0.072 (7)	0.053 (7)	0.056 (7)	0.005 (6)	0.019 (6)	0.015 (6)
C6	0.064 (7)	0.072 (8)	0.074 (8)	0.014 (6)	0.025 (6)	0.023 (7)
C7	0.060 (7)	0.078 (8)	0.071 (8)	0.003 (7)	0.013 (6)	0.024 (7)
C8	0.055 (7)	0.068 (8)	0.064 (7)	0.000 (6)	0.006 (6)	0.006 (6)
C9	0.093 (8)	0.062 (8)	0.080 (8)	−0.006 (7)	0.012 (7)	0.019 (7)
C10	0.067 (8)	0.075 (8)	0.069 (7)	−0.003 (7)	0.008 (6)	0.005 (7)
C11	0.059 (7)	0.046 (7)	0.049 (6)	−0.007 (6)	0.003 (5)	−0.001 (5)
C12	0.048 (6)	0.051 (7)	0.046 (6)	0.004 (5)	0.010 (5)	−0.001 (5)
C13	0.070 (8)	0.080 (8)	0.067 (8)	0.002 (7)	0.015 (6)	0.014 (7)
C14	0.090 (9)	0.108 (10)	0.093 (9)	0.014 (8)	0.035 (8)	0.011 (8)
C15	0.081 (8)	0.077 (8)	0.072 (8)	0.022 (7)	0.023 (7)	0.005 (7)
C16	0.076 (8)	0.063 (8)	0.066 (7)	0.015 (6)	0.023 (6)	0.016 (6)
C17	0.072 (7)	0.061 (8)	0.063 (7)	0.011 (6)	0.016 (6)	0.008 (6)
C18	0.075 (8)	0.059 (7)	0.067 (7)	−0.008 (6)	0.022 (6)	0.000 (6)
C19	0.053 (7)	0.053 (7)	0.057 (7)	−0.010 (6)	0.015 (6)	−0.006 (6)
C20	0.040 (6)	0.058 (7)	0.059 (7)	−0.007 (5)	0.016 (5)	−0.010 (6)
C21	0.078 (8)	0.074 (8)	0.090 (9)	−0.024 (7)	0.013 (7)	−0.005 (7)
C22	0.078 (8)	0.087 (9)	0.071 (8)	−0.022 (7)	0.004 (7)	−0.007 (7)
C23	0.076 (8)	0.084 (8)	0.067 (8)	−0.005 (7)	0.018 (6)	0.004 (7)
C24	0.055 (7)	0.062 (7)	0.058 (7)	−0.006 (6)	0.010 (6)	0.001 (6)
N1	0.055 (6)	0.062 (7)	0.063 (6)	−0.004 (5)	0.014 (5)	−0.004 (5)
N2	0.050 (5)	0.050 (6)	0.052 (6)	0.000 (5)	0.011 (5)	0.007 (5)
N3	0.062 (6)	0.055 (6)	0.055 (6)	−0.006 (5)	0.016 (5)	−0.002 (5)
N4	0.073 (7)	0.058 (7)	0.077 (7)	0.015 (6)	−0.002 (6)	−0.007 (6)
N5	0.059 (6)	0.046 (6)	0.056 (6)	0.010 (5)	0.000 (5)	0.003 (5)
N6	0.057 (6)	0.042 (6)	0.055 (6)	0.010 (5)	0.011 (5)	0.004 (5)
N7	0.060 (6)	0.078 (7)	0.075 (7)	−0.001 (6)	0.010 (6)	0.006 (6)
N8	0.040 (5)	0.061 (6)	0.053 (6)	−0.004 (5)	0.004 (5)	0.006 (5)
N9	0.056 (6)	0.058 (6)	0.060 (6)	−0.003 (5)	0.012 (5)	0.010 (5)
O1	0.058 (3)	0.057 (3)	0.058 (3)	−0.0004 (10)	0.0125 (11)	0.0000 (10)
O2	0.061 (3)	0.062 (3)	0.061 (3)	0.0006 (10)	0.0132 (11)	0.0009 (10)
O3	0.061 (3)	0.061 (3)	0.062 (3)	−0.0001 (10)	0.0139 (11)	0.0009 (10)
O4	0.051 (2)	0.052 (2)	0.052 (2)	0.0003 (10)	0.0108 (11)	−0.0007 (10)
O5	0.055 (2)	0.054 (2)	0.053 (2)	−0.0001 (10)	0.0118 (11)	−0.0002 (10)
O6	0.072 (6)	0.092 (6)	0.053 (5)	−0.038 (5)	0.019 (4)	−0.017 (5)
O7	0.086 (6)	0.056 (5)	0.061 (5)	−0.020 (5)	−0.026 (5)	0.019 (4)
O8	0.070 (5)	0.101 (6)	0.044 (5)	−0.038 (5)	0.017 (4)	−0.002 (5)
O9	0.066 (3)	0.066 (3)	0.067 (3)	0.0009 (10)	0.0142 (11)	−0.0014 (10)
O10	0.105 (7)	0.073 (6)	0.062 (6)	−0.049 (5)	0.018 (5)	−0.014 (5)
O11A	0.041 (8)	0.035 (8)	0.043 (8)	−0.001 (7)	0.010 (7)	0.005 (6)
O11B	0.028 (7)	0.030 (7)	0.038 (7)	0.003 (6)	0.000 (6)	0.002 (6)
O12	0.087 (6)	0.052 (5)	0.070 (6)	−0.028 (5)	−0.026 (5)	0.013 (5)
O13	0.092 (6)	0.076 (6)	0.053 (5)	0.050 (5)	−0.005 (5)	−0.017 (5)
O14	0.033 (7)	0.031 (7)	0.036 (7)	−0.003 (6)	0.007 (6)	0.007 (6)
O14A	0.042 (7)	0.039 (8)	0.044 (8)	0.003 (6)	0.002 (6)	−0.003 (6)
O15	0.093 (6)	0.059 (6)	0.138 (8)	0.002 (5)	0.069 (6)	−0.010 (6)
O16	0.035 (7)	0.038 (8)	0.036 (7)	0.008 (7)	0.009 (6)	0.000 (6)
O16B	0.023 (7)	0.033 (7)	0.029 (7)	0.004 (6)	0.008 (6)	−0.008 (6)
O17A	0.025 (6)	0.028 (6)	0.033 (6)	0.003 (5)	0.004 (5)	0.000 (5)
O17B	0.030 (6)	0.024 (6)	0.034 (6)	0.008 (5)	0.013 (5)	0.005 (5)
O18A	0.027 (6)	0.027 (6)	0.023 (6)	0.003 (5)	0.000 (5)	−0.003 (5)
O18B	0.030 (6)	0.023 (6)	0.028 (6)	0.001 (5)	0.001 (5)	−0.011 (5)
O19	0.101 (6)	0.094 (6)	0.060 (5)	0.044 (5)	0.006 (5)	0.001 (5)
O20	0.096 (8)	0.105 (9)	0.114 (9)	0.008 (7)	0.031 (7)	−0.007 (7)
O20A	0.040 (11)	0.048 (11)	0.036 (11)	0.020 (9)	0.007 (8)	0.000 (9)
O21A	0.024 (9)	0.043 (10)	0.031 (10)	−0.008 (8)	0.001 (8)	0.005 (8)
O21	0.079 (8)	0.096 (9)	0.111 (9)	0.003 (7)	−0.008 (7)	−0.001 (7)
O22	0.086 (6)	0.051 (6)	0.136 (7)	−0.005 (5)	0.062 (6)	−0.020 (5)

### Geometric parameters (Å, °)

**Table:**

Si1—O17A^i^	1.606 (13)	C1—H1	0.9300
Si1—O17A	1.606 (13)	C2—C3	1.410 (19)
Si1—O18A	1.648 (13)	C2—H2	0.9300
Si1—O18A^i^	1.648 (13)	C3—N8	1.325 (16)
Si1—O18B	1.670 (13)	C3—C4	1.455 (18)
Si1—O18B^i^	1.670 (13)	C4—N9	1.370 (16)
Si1—O17B	1.684 (13)	C4—C5	1.383 (18)
Si1—O17B^i^	1.684 (13)	C5—C6	1.409 (19)
Fe1—N2	2.105 (11)	C5—H5	0.9300
Fe1—N8	2.119 (11)	C6—C7	1.32 (2)
Fe1—N5	2.153 (11)	C6—H6	0.9300
Fe1—N3	2.173 (11)	C7—C8	1.38 (2)
Fe1—N9	2.186 (12)	C7—H7	0.9300
Fe1—N6	2.206 (11)	C8—N9	1.369 (17)
Mo1—O3	1.657 (9)	C8—H8	0.9300
Mo1—O7	1.823 (9)	C9—C10	1.37 (2)
Mo1—O19	1.832 (11)	C9—N4	1.368 (19)
Mo1—O16B	1.892 (16)	C9—H9	0.9300
Mo1—O11A	2.010 (19)	C10—C11	1.435 (19)
Mo1—O11B	2.117 (17)	C10—H10	0.9300
Mo1—O16	2.183 (17)	C11—N5	1.335 (16)
Mo1—O18B^i^	2.406 (13)	C11—C12	1.440 (18)
Mo1—O18A	2.423 (13)	C12—N6	1.332 (16)
Mo2—O5	1.646 (9)	C12—C13	1.417 (19)
Mo2—O7	1.923 (9)	C13—C14	1.38 (2)
Mo2—O12^i^	1.940 (10)	C13—H13	0.9300
Mo2—O15	1.950 (11)	C14—C15	1.38 (2)
Mo2—O22^i^	1.993 (10)	C14—H14	0.9300
Mo2—O17B	2.468 (13)	C15—C16	1.37 (2)
Mo2—O18A	2.498 (12)	C15—H15	0.9300
Mo3—O21	1.700 (15)	C16—N6	1.351 (17)
Mo3—O20A	1.72 (3)	C16—H16	0.9300
Mo3—O20	1.765 (17)	C17—N1	1.313 (17)
Mo3—O21A	1.84 (3)	C17—C18	1.349 (19)
Mo3—O16	1.998 (16)	C17—H17	0.9300
Mo3—O10	2.033 (9)	C18—C19	1.444 (19)
Mo3—O11B	2.051 (17)	C18—H18	0.9300
Mo3—O11A	2.086 (19)	C19—N2	1.333 (16)
Mo3—O14A	2.099 (16)	C19—C20	1.452 (18)
Mo3—O14	2.164 (15)	C20—N3	1.378 (16)
Mo3—O16B	2.174 (15)	C20—C21	1.411 (19)
Mo4—O4	1.663 (8)	C21—C22	1.39 (2)
Mo4—O22	1.803 (10)	C21—H21	0.9300
Mo4—O13	1.839 (9)	C22—C23	1.37 (2)
Mo4—O14	1.895 (15)	C22—H22	0.9300
Mo4—O11A	1.903 (19)	C23—C24	1.37 (2)
Mo4—O11B	2.228 (18)	C23—H23	0.9300
Mo4—O14A	2.237 (17)	C24—N3	1.335 (16)
Mo4—O17B^i^	2.397 (14)	C24—H24	0.9300
Mo4—O18B^i^	2.435 (13)	N1—N2	1.359 (14)
Mo5—O1	1.659 (9)	N1—H1A	0.8600
Mo5—O19	1.918 (11)	N4—N5	1.382 (15)
Mo5—O8	1.928 (9)	N4—H4	0.8600
Mo5—O6	1.952 (9)	N7—N8	1.339 (14)
Mo5—O13	1.978 (9)	N7—H7A	0.8600
Mo5—O18B^i^	2.404 (13)	O10—Mo7^i^	2.058 (10)
Mo5—O17A	2.463 (14)	O10—Mo6^i^	2.061 (11)
Mo6—O2	1.668 (9)	O11A—O11B	0.701 (18)
Mo6—O8	1.833 (9)	O12—Mo2^i^	1.940 (10)
Mo6—O15	1.832 (10)	O14—O14A	0.878 (17)
Mo6—O14^i^	1.954 (15)	O14—Mo6^i^	1.954 (15)
Mo6—O10^i^	2.061 (11)	O14A—Mo6^i^	2.119 (17)
Mo6—O14A^i^	2.119 (17)	O16—O16B	0.766 (16)
Mo6—O17B	2.375 (13)	O16—Mo7^i^	2.154 (17)
Mo6—O17A	2.480 (13)	O16B—Mo7^i^	1.943 (16)
Mo7—O9	1.664 (10)	O17A—O18B^i^	1.778 (18)
Mo7—O12	1.818 (9)	O17B—Mo4^i^	2.397 (14)
Mo7—O6	1.840 (9)	O18A—Mo7^i^	2.389 (13)
Mo7—O16B^i^	1.943 (16)	O18B—O17A^i^	1.778 (18)
Mo7—O10^i^	2.058 (10)	O18B—Mo5^i^	2.404 (13)
Mo7—O16^i^	2.154 (17)	O18B—Mo1^i^	2.406 (13)
Mo7—O18A^i^	2.389 (13)	O18B—Mo4^i^	2.435 (13)
Mo7—O17A	2.417 (13)	O20—O20A	1.79 (4)
C1—C2	1.34 (2)	O21A—O21	1.68 (3)
C1—N7	1.337 (19)	O22—Mo2^i^	1.993 (10)
			
O17A^i^—Si1—O17A	180.0 (10)	O14^i^—Mo6—O14A^i^	24.5 (5)
O17A^i^—Si1—O18A	69.8 (7)	O10^i^—Mo6—O14A^i^	74.5 (5)
O17A—Si1—O18A	110.2 (7)	O2—Mo6—O17B	154.3 (5)
O17A^i^—Si1—O18A^i^	110.2 (7)	O8—Mo6—O17B	101.6 (5)
O17A—Si1—O18A^i^	69.8 (7)	O15—Mo6—O17B	65.6 (5)
O18A—Si1—O18A^i^	180.000 (3)	O14^i^—Mo6—O17B	49.8 (6)
O17A^i^—Si1—O18B	65.7 (6)	O10^i^—Mo6—O17B	91.8 (4)
O17A—Si1—O18B	114.3 (6)	O14A^i^—Mo6—O17B	72.4 (6)
O18A—Si1—O18B	108.4 (6)	O2—Mo6—O17A	156.3 (4)
O18A^i^—Si1—O18B	71.6 (6)	O8—Mo6—O17A	67.0 (4)
O17A^i^—Si1—O18B^i^	114.3 (6)	O15—Mo6—O17A	101.0 (5)
O17A—Si1—O18B^i^	65.7 (6)	O14^i^—Mo6—O17A	76.8 (6)
O18A—Si1—O18B^i^	71.6 (6)	O10^i^—Mo6—O17A	63.1 (4)
O18A^i^—Si1—O18B^i^	108.4 (6)	O14A^i^—Mo6—O17A	100.1 (6)
O18B—Si1—O18B^i^	180.0 (6)	O17B—Mo6—O17A	47.2 (5)
O17A^i^—Si1—O17B	107.4 (7)	O9—Mo7—O12	102.6 (5)
O17A—Si1—O17B	72.6 (7)	O9—Mo7—O6	100.4 (5)
O18A—Si1—O17B	68.4 (6)	O12—Mo7—O6	97.2 (4)
O18A^i^—Si1—O17B	111.6 (6)	O9—Mo7—O16B^i^	111.2 (6)
O18B—Si1—O17B	75.3 (6)	O12—Mo7—O16B^i^	88.3 (5)
O18B^i^—Si1—O17B	104.7 (6)	O6—Mo7—O16B^i^	145.8 (6)
O17A^i^—Si1—O17B^i^	72.6 (7)	O9—Mo7—O10^i^	97.4 (5)
O17A—Si1—O17B^i^	107.4 (7)	O12—Mo7—O10^i^	156.2 (5)
O18A—Si1—O17B^i^	111.6 (6)	O6—Mo7—O10^i^	91.6 (4)
O18A^i^—Si1—O17B^i^	68.4 (6)	O16B^i^—Mo7—O10^i^	72.4 (5)
O18B—Si1—O17B^i^	104.7 (6)	O9—Mo7—O16^i^	90.5 (6)
O18B^i^—Si1—O17B^i^	75.3 (6)	O12—Mo7—O16^i^	94.0 (5)
O17B—Si1—O17B^i^	180.000 (3)	O6—Mo7—O16^i^	162.2 (5)
N2—Fe1—N8	102.7 (4)	O16B^i^—Mo7—O16^i^	20.7 (5)
N2—Fe1—N5	161.1 (4)	O10^i^—Mo7—O16^i^	72.9 (5)
N8—Fe1—N5	89.3 (4)	O9—Mo7—O18A^i^	156.7 (5)
N2—Fe1—N3	76.0 (4)	O12—Mo7—O18A^i^	66.4 (5)
N8—Fe1—N3	166.1 (4)	O6—Mo7—O18A^i^	101.3 (5)
N5—Fe1—N3	95.8 (4)	O16B^i^—Mo7—O18A^i^	50.4 (6)
N2—Fe1—N9	96.8 (4)	O10^i^—Mo7—O18A^i^	90.3 (4)
N8—Fe1—N9	75.2 (4)	O16^i^—Mo7—O18A^i^	70.7 (5)
N5—Fe1—N9	100.5 (4)	O9—Mo7—O17A	155.7 (5)
N3—Fe1—N9	91.1 (4)	O12—Mo7—O17A	99.3 (5)
N2—Fe1—N6	90.3 (4)	O6—Mo7—O17A	66.2 (5)
N8—Fe1—N6	92.0 (4)	O16B^i^—Mo7—O17A	79.6 (6)
N5—Fe1—N6	74.5 (4)	O10^i^—Mo7—O17A	64.3 (5)
N3—Fe1—N6	101.8 (4)	O16^i^—Mo7—O17A	98.4 (6)
N9—Fe1—N6	166.5 (4)	O18A^i^—Mo7—O17A	45.6 (4)
O3—Mo1—O7	101.6 (5)	C2—C1—N7	110.5 (15)
O3—Mo1—O19	102.0 (5)	C2—C1—H1	124.7
O7—Mo1—O19	95.9 (5)	N7—C1—H1	124.8
O3—Mo1—O16B	111.0 (6)	C1—C2—C3	102.9 (15)
O7—Mo1—O16B	89.0 (5)	C1—C2—H2	128.5
O19—Mo1—O16B	144.9 (6)	C3—C2—H2	128.6
O3—Mo1—O11A	108.1 (6)	N8—C3—C2	111.1 (13)
O7—Mo1—O11A	147.9 (6)	N8—C3—C4	117.5 (13)
O19—Mo1—O11A	89.7 (6)	C2—C3—C4	131.4 (14)
O16B—Mo1—O11A	69.4 (7)	N9—C4—C5	121.5 (13)
O3—Mo1—O11B	88.8 (6)	N9—C4—C3	114.1 (12)
O7—Mo1—O11B	165.0 (6)	C5—C4—C3	124.4 (13)
O19—Mo1—O11B	92.5 (6)	C4—C5—C6	118.5 (14)
O16B—Mo1—O11B	77.0 (6)	C4—C5—H5	120.7
O11A—Mo1—O11B	19.3 (5)	C6—C5—H5	120.8
O3—Mo1—O16	90.9 (6)	C7—C6—C5	119.9 (15)
O7—Mo1—O16	93.6 (5)	C7—C6—H6	120.1
O19—Mo1—O16	162.0 (6)	C5—C6—H6	120.0
O16B—Mo1—O16	20.1 (5)	C6—C7—C8	120.6 (16)
O11A—Mo1—O16	74.4 (7)	C6—C7—H7	119.6
O11B—Mo1—O16	75.2 (6)	C8—C7—H7	119.8
O3—Mo1—O18B^i^	154.5 (4)	N9—C8—C7	121.8 (15)
O7—Mo1—O18B^i^	100.7 (5)	N9—C8—H8	119.1
O19—Mo1—O18B^i^	63.6 (5)	C7—C8—H8	119.1
O16B—Mo1—O18B^i^	81.4 (6)	C10—C9—N4	106.4 (14)
O11A—Mo1—O18B^i^	54.1 (6)	C10—C9—H9	126.8
O11B—Mo1—O18B^i^	71.9 (6)	N4—C9—H9	126.7
O16—Mo1—O18B^i^	99.7 (5)	C9—C10—C11	105.6 (14)
O3—Mo1—O18A	156.2 (5)	C9—C10—H10	127.1
O7—Mo1—O18A	67.5 (4)	C11—C10—H10	127.2
O19—Mo1—O18A	100.2 (5)	N5—C11—C10	111.2 (13)
O16B—Mo1—O18A	50.1 (6)	N5—C11—C12	117.7 (12)
O11A—Mo1—O18A	80.4 (6)	C10—C11—C12	131.1 (13)
O11B—Mo1—O18A	98.8 (6)	N6—C12—C13	122.3 (13)
O16—Mo1—O18A	69.6 (5)	N6—C12—C11	115.7 (11)
O18B^i^—Mo1—O18A	47.4 (4)	C13—C12—C11	121.9 (13)
O5—Mo2—O7	102.7 (4)	C14—C13—C12	118.5 (16)
O5—Mo2—O12^i^	103.1 (4)	C14—C13—H13	120.7
O7—Mo2—O12^i^	88.3 (4)	C12—C13—H13	120.7
O5—Mo2—O15	101.1 (5)	C13—C14—C15	117.6 (17)
O7—Mo2—O15	89.7 (5)	C13—C14—H14	121.1
O12^i^—Mo2—O15	155.6 (5)	C15—C14—H14	121.3
O5—Mo2—O22^i^	101.5 (5)	C16—C15—C14	121.8 (17)
O7—Mo2—O22^i^	155.8 (5)	C16—C15—H15	119.2
O12^i^—Mo2—O22^i^	85.7 (4)	C14—C15—H15	119.1
O15—Mo2—O22^i^	86.2 (4)	N6—C16—C15	121.1 (15)
O5—Mo2—O17B	155.9 (4)	N6—C16—H16	119.5
O7—Mo2—O17B	94.9 (5)	C15—C16—H16	119.4
O12^i^—Mo2—O17B	93.7 (4)	N1—C17—C18	109.6 (14)
O15—Mo2—O17B	62.2 (5)	N1—C17—H17	125.2
O22^i^—Mo2—O17B	62.2 (4)	C18—C17—H17	125.2
O5—Mo2—O18A	159.8 (4)	C17—C18—C19	102.8 (14)
O7—Mo2—O18A	64.6 (4)	C17—C18—H18	128.7
O12^i^—Mo2—O18A	62.6 (4)	C19—C18—H18	128.5
O15—Mo2—O18A	94.8 (5)	N2—C19—C18	111.2 (12)
O22^i^—Mo2—O18A	92.0 (5)	N2—C19—C20	116.9 (12)
O17B—Mo2—O18A	44.3 (4)	C18—C19—C20	131.9 (13)
O21—Mo3—O20A	66.1 (11)	N3—C20—C21	120.6 (13)
O21—Mo3—O20	97.1 (8)	N3—C20—C19	114.9 (11)
O20A—Mo3—O20	61.9 (12)	C21—C20—C19	124.5 (14)
O21—Mo3—O21A	56.4 (10)	C22—C21—C20	116.7 (16)
O20A—Mo3—O21A	92.6 (14)	C22—C21—H21	121.6
O20—Mo3—O21A	66.6 (11)	C20—C21—H21	121.8
O21—Mo3—O16	161.4 (8)	C23—C22—C21	121.5 (16)
O20A—Mo3—O16	104.3 (11)	C23—C22—H22	119.2
O20—Mo3—O16	64.5 (7)	C21—C22—H22	119.2
O21A—Mo3—O16	110.4 (10)	C24—C23—C22	119.2 (16)
O21—Mo3—O10	115.0 (7)	C24—C23—H23	120.4
O20A—Mo3—O10	79.4 (11)	C22—C23—H23	120.4
O20—Mo3—O10	113.0 (7)	N3—C24—C23	121.5 (14)
O21A—Mo3—O10	170.5 (9)	N3—C24—H24	119.2
O16—Mo3—O10	76.8 (6)	C23—C24—H24	119.2
O21—Mo3—O11B	102.1 (7)	C17—N1—N2	112.8 (12)
O20A—Mo3—O11B	157.2 (12)	C17—N1—H1A	123.5
O20—Mo3—O11B	102.4 (8)	N2—N1—H1A	123.7
O21A—Mo3—O11B	65.1 (10)	C19—N2—N1	103.6 (11)
O16—Mo3—O11B	80.8 (7)	C19—N2—Fe1	117.6 (9)
O10—Mo3—O11B	123.3 (6)	N1—N2—Fe1	138.7 (9)
O21—Mo3—O11A	111.9 (8)	C24—N3—C20	120.2 (12)
O20A—Mo3—O11A	176.6 (12)	C24—N3—Fe1	125.0 (10)
O20—Mo3—O11A	116.3 (8)	C20—N3—Fe1	114.6 (8)
O21A—Mo3—O11A	84.1 (10)	C9—N4—N5	112.5 (13)
O16—Mo3—O11A	76.8 (7)	C9—N4—H4	123.8
O10—Mo3—O11A	104.0 (6)	N5—N4—H4	123.7
O11B—Mo3—O11A	19.5 (5)	C11—N5—N4	104.1 (12)
O21—Mo3—O14A	65.6 (8)	C11—N5—Fe1	115.4 (9)
O20A—Mo3—O14A	107.0 (11)	N4—N5—Fe1	138.9 (10)
O20—Mo3—O14A	162.7 (7)	C12—N6—C16	118.5 (12)
O21A—Mo3—O14A	102.4 (10)	C12—N6—Fe1	114.9 (8)
O16—Mo3—O14A	132.9 (7)	C16—N6—Fe1	125.8 (10)
O10—Mo3—O14A	75.5 (5)	C1—N7—N8	109.7 (13)
O11B—Mo3—O14A	83.6 (7)	C1—N7—H7A	125.1
O11A—Mo3—O14A	74.0 (7)	N8—N7—H7A	125.2
O21—Mo3—O14	88.8 (7)	C3—N8—N7	105.7 (12)
O20A—Mo3—O14	124.0 (11)	C3—N8—Fe1	117.6 (9)
O20—Mo3—O14	173.2 (7)	N7—N8—Fe1	136.6 (10)
O21A—Mo3—O14	114.7 (10)	C8—N9—C4	117.6 (12)
O16—Mo3—O14	109.4 (7)	C8—N9—Fe1	126.9 (10)
O10—Mo3—O14	66.9 (5)	C4—N9—Fe1	115.4 (8)
O11B—Mo3—O14	73.0 (6)	Mo7—O6—Mo5	137.3 (6)
O11A—Mo3—O14	58.0 (6)	Mo1—O7—Mo2	137.5 (6)
O14A—Mo3—O14	23.7 (5)	Mo6—O8—Mo5	136.7 (5)
O21—Mo3—O16B	174.7 (7)	Mo3—O10—Mo7^i^	106.3 (4)
O20A—Mo3—O16B	119.1 (11)	Mo3—O10—Mo6^i^	107.2 (4)
O20—Mo3—O16B	84.9 (7)	Mo7^i^—O10—Mo6^i^	129.4 (6)
O21A—Mo3—O16B	120.5 (9)	O11B—O11A—Mo4	109 (3)
O16—Mo3—O16B	20.6 (5)	O11B—O11A—Mo1	89 (3)
O10—Mo3—O16B	68.3 (5)	Mo4—O11A—Mo1	143.0 (10)
O11B—Mo3—O16B	72.6 (6)	O11B—O11A—Mo3	77 (2)
O11A—Mo3—O16B	62.9 (7)	Mo4—O11A—Mo3	111.0 (9)
O14A—Mo3—O16B	112.4 (6)	Mo1—O11A—Mo3	104.4 (8)
O14—Mo3—O16B	88.8 (6)	O11A—O11B—Mo3	83 (2)
O4—Mo4—O22	101.8 (5)	O11A—O11B—Mo1	72 (2)
O4—Mo4—O13	100.9 (5)	Mo3—O11B—Mo1	101.9 (7)
O22—Mo4—O13	96.4 (5)	O11A—O11B—Mo4	54 (2)
O4—Mo4—O14	110.5 (6)	Mo3—O11B—Mo4	100.4 (7)
O22—Mo4—O14	91.7 (6)	Mo1—O11B—Mo4	117.2 (7)
O13—Mo4—O14	145.2 (6)	Mo7—O12—Mo2^i^	138.9 (6)
O4—Mo4—O11A	106.1 (6)	Mo4—O13—Mo5	135.4 (6)
O22—Mo4—O11A	148.9 (7)	O14A—O14—Mo4	101.1 (17)
O13—Mo4—O11A	91.8 (6)	O14A—O14—Mo6^i^	88.3 (16)
O14—Mo4—O11A	65.8 (7)	Mo4—O14—Mo6^i^	145.8 (9)
O4—Mo4—O11B	89.0 (5)	O14A—O14—Mo3	73.9 (15)
O22—Mo4—O11B	164.8 (6)	Mo4—O14—Mo3	108.0 (7)
O13—Mo4—O11B	91.9 (5)	Mo6^i^—O14—Mo3	106.1 (7)
O14—Mo4—O11B	74.4 (6)	O14—O14A—Mo3	82.4 (16)
O11A—Mo4—O11B	17.4 (6)	O14—O14A—Mo6^i^	67.2 (15)
O4—Mo4—O14A	88.1 (5)	Mo3—O14A—Mo6^i^	102.7 (7)
O22—Mo4—O14A	93.1 (5)	O14—O14A—Mo4	56.2 (15)
O13—Mo4—O14A	165.3 (5)	Mo3—O14A—Mo4	98.6 (7)
O14—Mo4—O14A	22.7 (5)	Mo6^i^—O14A—Mo4	115.3 (8)
O11A—Mo4—O14A	74.4 (7)	Mo6—O15—Mo2	138.4 (7)
O11B—Mo4—O14A	76.5 (6)	O16B—O16—Mo3	92.8 (19)
O4—Mo4—O17B^i^	153.6 (4)	O16B—O16—Mo7^i^	63.9 (19)
O22—Mo4—O17B^i^	66.0 (5)	Mo3—O16—Mo7^i^	104.0 (7)
O13—Mo4—O17B^i^	103.7 (5)	O16B—O16—Mo1	58.0 (18)
O14—Mo4—O17B^i^	49.8 (6)	Mo3—O16—Mo1	101.4 (7)
O11A—Mo4—O17B^i^	82.9 (6)	Mo7^i^—O16—Mo1	116.9 (8)
O11B—Mo4—O17B^i^	99.7 (5)	O16—O16B—Mo1	102 (2)
O14A—Mo4—O17B^i^	70.0 (5)	O16—O16B—Mo7^i^	95 (2)
O4—Mo4—O18B^i^	153.7 (4)	Mo1—O16B—Mo7^i^	149.0 (9)
O22—Mo4—O18B^i^	102.3 (5)	O16—O16B—Mo3	66.6 (18)
O13—Mo4—O18B^i^	66.0 (4)	Mo1—O16B—Mo3	105.3 (7)
O14—Mo4—O18B^i^	79.3 (6)	Mo7^i^—O16B—Mo3	105.2 (7)
O11A—Mo4—O18B^i^	54.4 (6)	Si1—O17A—O18B^i^	58.9 (6)
O11B—Mo4—O18B^i^	69.6 (5)	Si1—O17A—Mo7	122.6 (7)
O14A—Mo4—O18B^i^	101.0 (5)	O18B^i^—O17A—Mo7	130.3 (8)
O17B^i^—Mo4—O18B^i^	50.1 (5)	Si1—O17A—Mo5	125.7 (7)
O1—Mo5—O19	100.9 (5)	O18B^i^—O17A—Mo5	66.8 (6)
O1—Mo5—O8	101.2 (4)	Mo7—O17A—Mo5	92.7 (5)
O19—Mo5—O8	90.3 (4)	Si1—O17A—Mo6	119.1 (7)
O1—Mo5—O6	101.8 (4)	O18B^i^—O17A—Mo6	124.3 (8)
O19—Mo5—O6	157.2 (5)	Mo7—O17A—Mo6	99.0 (5)
O8—Mo5—O6	87.2 (4)	Mo5—O17A—Mo6	90.0 (4)
O1—Mo5—O13	99.9 (4)	Si1—O17B—Mo6	121.1 (7)
O19—Mo5—O13	87.1 (4)	Si1—O17B—Mo4^i^	117.9 (6)
O8—Mo5—O13	158.8 (4)	Mo6—O17B—Mo4^i^	100.9 (5)
O6—Mo5—O13	87.2 (4)	Si1—O17B—Mo2	123.6 (6)
O1—Mo5—O18B^i^	156.9 (4)	Mo6—O17B—Mo2	93.7 (4)
O19—Mo5—O18B^i^	62.6 (5)	Mo4^i^—O17B—Mo2	93.6 (5)
O8—Mo5—O18B^i^	95.3 (4)	Si1—O18A—Mo7^i^	122.1 (7)
O6—Mo5—O18B^i^	95.0 (5)	Si1—O18A—Mo1	120.6 (7)
O13—Mo5—O18B^i^	64.9 (4)	Mo7^i^—O18A—Mo1	100.3 (5)
O1—Mo5—O17A	160.3 (4)	Si1—O18A—Mo2	123.7 (6)
O19—Mo5—O17A	94.5 (5)	Mo7^i^—O18A—Mo2	92.1 (4)
O8—Mo5—O17A	66.2 (4)	Mo1—O18A—Mo2	90.4 (4)
O6—Mo5—O17A	63.8 (4)	Si1—O18B—O17A^i^	55.4 (6)
O13—Mo5—O17A	93.0 (5)	Si1—O18B—Mo5^i^	125.8 (7)
O18B^i^—Mo5—O17A	42.8 (4)	O17A^i^—O18B—Mo5^i^	70.4 (6)
O2—Mo6—O8	101.7 (5)	Si1—O18B—Mo1^i^	120.5 (6)
O2—Mo6—O15	101.0 (5)	O17A^i^—O18B—Mo1^i^	132.3 (8)
O8—Mo6—O15	96.4 (5)	Mo5^i^—O18B—Mo1^i^	94.1 (5)
O2—Mo6—O14^i^	111.8 (6)	Si1—O18B—Mo4^i^	116.6 (7)
O8—Mo6—O14^i^	143.8 (6)	O17A^i^—O18B—Mo4^i^	125.0 (8)
O15—Mo6—O14^i^	90.5 (6)	Mo5^i^—O18B—Mo4^i^	93.7 (4)
O2—Mo6—O10^i^	98.0 (5)	Mo1^i^—O18B—Mo4^i^	100.1 (5)
O8—Mo6—O10^i^	92.2 (4)	Mo1—O19—Mo5	139.7 (7)
O15—Mo6—O10^i^	157.0 (5)	Mo3—O20—O20A	57.8 (11)
O14^i^—Mo6—O10^i^	70.5 (5)	Mo3—O20A—O20	60.3 (11)
O2—Mo6—O14A^i^	87.4 (6)	O21—O21A—Mo3	57.7 (10)
O8—Mo6—O14A^i^	165.0 (5)	O21A—O21—Mo3	65.9 (11)
O15—Mo6—O14A^i^	93.6 (6)	Mo4—O22—Mo2^i^	138.2 (6)

Symmetry codes: (i) −*x*, −*y*+1, −*z*+1.

### Hydrogen-bond geometry (Å, °)

**Table:**

*D*—H···*A*	*D*—H	H···*A*	*D*···*A*	*D*—H···*A*
N4—H4···O13^ii^	0.86	2.15	2.973 (17)	159
N1—H1A···O21A^iii^	0.86	2.17	2.96 (3)	152
N1—H1A···O21^iii^	0.86	2.05	2.844 (19)	153

Symmetry codes: (ii) *x*, −*y*+3/2, *z*−1/2; (iii) −*x*+1, −*y*+1, −*z*+1.

## References

[bb1] Brese, N. E. & O’Keeffe, M. (1991). *Acta Cryst.* B**47**, 192–197.

[bb2] Bruker (2001). *SAINT-Plus* and *SADABS* Bruker AXS Inc., Madison, Wisconsin, USA.

[bb3] Bruker (2004). *APEX2* Bruker AXS Inc., Madison, Wisconsin, USA.

[bb4] Dolbecq, A., Cadot, E., Eisner, D. & Secheresse, F. (1999). *Inorg. Chem.* **38**, 4127–4134.

[bb5] Pope, M. T. & Müller, A. (1991). *Angew. Chem. Int. Ed.* **30**, 34–38.

[bb6] Sheldrick, G. M. (2008). *Acta Cryst.* A**64**, 112–122. 10.1107/S0108767307043930 18156677

[bb7] Yang, W. B., Lu, C. Z., Wu, C. D. & Zhuang, H. H. (2003). *Chin. J. Struct. Chem.* **22**, 137–142.

[bb8] Zhang, X. T., Dou, J. M., Wei, P. H., Li, D. C., Li, B., Shi, C. W. & Hu, B. (2009). *Inorg. Chim. Acta*, **362**, 3325–3332.

[bb9] Zhang, X., Wei, P., Shi, C., Li, B. & Hu, B. (2010*a*). *Acta Cryst.* E**66**, m26–m27. 10.1107/S1600536809052003 PMC2980048 21579926

[bb13] Zhang, X., Wei, P., Shi, C., Li, B. & Hu, B. (2010*b*). *Acta Cryst.* E**66**, m174–m175. 10.1107/S160053681000156X PMC2979923 21579647

[bb10] Zhang, X. T., Wei, P. H., Sun, D. F., Ni, Z. H., Dou, J. M., Li, B., Shi, C. W. & Hu, B. (2009). *Cryst. Growth Des.* **9**, 4424–4428.

[bb11] Zhang, X., Wei, P., Zhu, W., Li, B. & Hu, B. (2010). *Acta Cryst.* E**66**, m127–m128. 10.1107/S160053681000019X PMC2979865 21579612

[bb12] Zhang, X., Yuan, D., Wei, P., Li, B. & Hu, B. (2010). *Acta Cryst.* E**66**, m152–m153. 10.1107/S1600536810000978 PMC2979803 21579630

